# 1*H*-Indole-3-carbaldehyde azine

**DOI:** 10.1107/S1600536808003164

**Published:** 2008-02-06

**Authors:** Mohd. Razali Rizal, Hapipah M. Ali, Seik Weng Ng

**Affiliations:** a Department of Chemistry, University of Malaya, 50603 Kuala Lumpur, Malaysia

## Abstract

The molecule of the title compound, C_18_H_14_N_4_, lies on a center of inversion such that there is one half-mol­ecule in the asymmetric unit. The N—N single bond adopts a *trans* configuration and the indole fused-ring system is nearly coplanar with the –CH=N—N=CH– fragment [dihedral angle = 9.8 (2)°]. Adjacent mol­ecules are linked by indole–azine N—H⋯N hydrogen bonds into a layer motif.

## Related literature

For the synthesis, see: Alemany *et al.* (1970 ▶); Swaminathan & Narasimhan (1964 ▶). For the crystal structures of some aromatic azines, for example, benzalazine, see: Burke-Laing & Laing (1976 ▶); Mom & de With (1978 ▶); Sinha, 1970 ▶). For other heterocyclic aldehyde azines, see: Lin *et al.* (2001*a* ▶,*b* ▶); Wu *et al.* (2006 ▶).
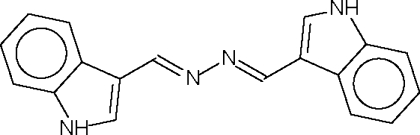


## Experimental

### 

#### Crystal data

C_18_H_14_N_4_
*M* *_r_* = 286.33
Monoclinic, 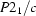

*a* = 5.0849 (2) Å
*b* = 10.6708 (4) Å
*c* = 13.4435 (5) Å
β = 94.366 (3)°
*V* = 727.33 (5) Å^3^
*Z* = 2
Mo *K*α radiation
μ = 0.08 mm^−1^
*T* = 295 (2) K
0.33 × 0.27 × 0.17 mm

#### Data collection

Bruker APEX2 diffractometer
Absorption correction: none
5388 measured reflections
1659 independent reflections
1085 reflections with *I* > 2σ(*I*)
*R*_int_ = 0.038

#### Refinement

*R*[*F* ^2^ > 2σ(*F* ^2^)] = 0.042
*wR*(*F* ^2^) = 0.121
*S* = 1.01
1659 reflections
105 parameters
H atoms treated by a mixture of independent and constrained refinement
Δρ_max_ = 0.17 e Å^−3^
Δρ_min_ = −0.16 e Å^−3^

### 

Data collection: *APEX2* (Bruker, 2007 ▶); cell refinement: *SAINT* (Bruker, 2007 ▶); data reduction: *SAINT*; program(s) used to solve structure: *SHELXS97* (Sheldrick, 2008 ▶); program(s) used to refine structure: *SHELXL97* (Sheldrick, 2008 ▶); molecular graphics: *X-SEED* (Barbour, 2001 ▶); software used to prepare material for publication: *publCIF* (Westrip, 2008 ▶).

## Supplementary Material

Crystal structure: contains datablocks global, I. DOI: 10.1107/S1600536808003164/fl2186sup1.cif

Structure factors: contains datablocks I. DOI: 10.1107/S1600536808003164/fl2186Isup2.hkl

Additional supplementary materials:  crystallographic information; 3D view; checkCIF report

## Figures and Tables

**Table 1:** Hydrogen-bond geometry (Å, °)

*D*—H⋯*A*	*D*—H	H⋯*A*	*D*⋯*A*	*D*—H⋯*A*
N1—H1⋯N2^i^	0.87 (2)	2.21 (2)	3.065 (2)	167 (2)

Symmetry code: (i) 
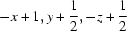
.

**Fig. 1.**
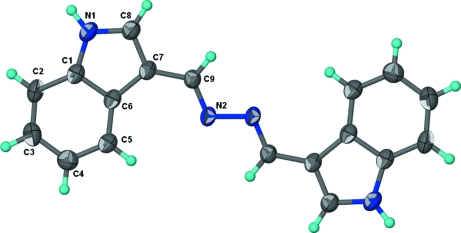
Displacement ellipsoid plot of (I) at the 50% probability level. H atoms are drawn as spheres of arbitrary radiius.

**Fig. 2.**
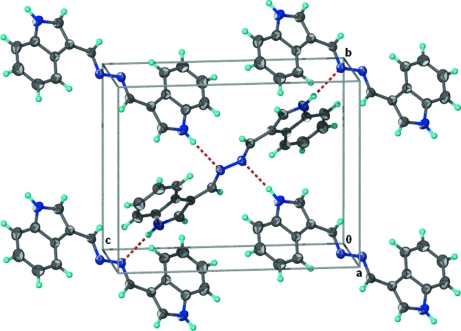
Layer structure of (I).

## supplementary crystallographic information

### Comment

Azines are readily synthesized by condensing hydrazine with an aldehyde; the crystal structures of a large number of substituted benzaldehdye azines have been reported. The structure of the parent aromatic compound, benzalazine, has been known for a long time (Burke-Laing & Laing, 1976; Mom & de With, 1978; Sinha, 1970). There are few examples of heterocyclic azines, and their rarity can be attributed to the difficulty of synthesizing the starting aldehyde reactant. Among the few are, for example, unsubstituted and methyl-subsituted thiophene-2-aldehyde azine (Lin *et al.*, 2001*a*, 2001*b*) and a pyrrole derivative has recently been reported (Wu *et al.*, 2006).

3-Indole azine has been known for some time; it was first synthesized from indole-3-carboxaldehyde and hydrazine in order to examine its psychopharmacological activity (Alemany *et al.*, 1970; Swaminathan Narasimhan, 1964). The title compound was the unexpected decomposition product of the Schiff base derived from the condensation of carbohydrazide and indole-3-carboxaldehyde. The molecule (Scheme I, Fig. 1) lies about a center-of-inversion such that there is half a molecule in the asymmetric unit. The N–N single-bond adopts a *trans* configuration and the indolyl fused-ring is nearly coplanar with the –CH=N–N=CH– fragment. Adjacent molecules are linked by an *N*–H_indole_···N_azine_ hydrogen bonds into layer motif (Fig. 2).

### Experimental

The reaction of carbohydrazide (0.3 g, 3.3 mmol) and indole -3-carboxaldehyde (1 g, 6.6 mmol) in ethanol under reflux for 2 h gave the corresponding Schiff base. This compound (0.2 g, 0.6 mmol), zinc acetate (0.06 g,0.3 mmol) and several drops of triethylamine were dissolved in 10 ml e thanol. The contents were heated in a 25-ml, stainless-steel Paar bomb for for 2 d at 373 K. The bomb was cooled to room temperature over several hours. Well formed crystals were isolated from the cooled bomb.

### Refinement

Carbon-bound H-atoms were placed in calculated positions (C—H 0.93 Å) and were included in the refinement in the riding model approximation, with *U*(H) set to 1.2*U*(C). The amino H-atom was located in a difference Fourier map, and was freely refined.

### Crystal data

**Table:**

C_18_H_14_N_4_	*F*_000_ = 300
*M**_r_* = 286.33	*D*_x_ = 1.307 Mg m^−^^3^
Monoclinic, *P*2_1_/*c*	Mo *K*α radiation λ = 0.71073 Å
Hall symbol: -P 2ybc	Cell parameters from 1012 reflections
*a* = 5.0849 (2) Å	θ = 2.3–23.6º
*b* = 10.6708 (4) Å	µ = 0.08 mm^−^^1^
*c* = 13.4435 (5) Å	*T* = 295 (2) K
β = 94.366 (3)º	Irregular block, green–yellow
*V* = 727.33 (5) Å^3^	0.33 × 0.27 × 0.17 mm
*Z* = 2	

### Data collection

**Table:**

Bruker APEX2 diffractometer	1085 reflections with *I* > 2σ(*I*)
Radiation source: fine-focus sealed tube	*R*_int_ = 0.038
Monochromator: graphite	θ_max_ = 27.5º
*T* = 295(2) K	θ_min_ = 2.4º
φ and ω scans	*h* = −6→6
Absorption correction: none	*k* = −9→13
5388 measured reflections	*l* = −17→17
1659 independent reflections	

### Refinement

**Table:**

Refinement on *F*^2^	Hydrogen site location: inferred from neighbouring sites
Least-squares matrix: full	H atoms treated by a mixture of independent and constrained refinement
*R*[*F*^2^ > 2σ(*F*^2^)] = 0.042	*w* = 1/[σ^2^(*F*_o_^2^) + (0.0645*P*)^2^ + ] where *P* = (*F*_o_^2^ + 2*F*_c_^2^)/3
*wR*(*F*^2^) = 0.121	(Δ/σ)_max_ = 0.001
*S* = 1.01	Δρ_max_ = 0.17 e Å^−^^3^
1659 reflections	Δρ_min_ = −0.16 e Å^−^^3^
105 parameters	Extinction correction: SHELXL97 (Sheldrick, 2008), Fc^*^=kFc[1+0.001xFc^2^λ^3^/sin(2θ)]^-1/4^
Primary atom site location: structure-invariant direct methods	Extinction coefficient: 0.016 (6)
Secondary atom site location: difference Fourier map	

### Fractional atomic coordinates and isotropic or equivalent isotropic displacement parameters (Å^2^)

**Table:**

	*x*	*y*	*z*	*U*_iso_*/*U*_eq_	
N1	0.4730 (3)	0.8059 (1)	0.1978 (1)	0.0484 (4)	
N2	0.4464 (2)	0.5165 (1)	0.4519 (1)	0.0422 (4)	
C1	0.2718 (3)	0.7222 (1)	0.1726 (1)	0.0425 (4)	
C2	0.1064 (3)	0.7130 (2)	0.0860 (1)	0.0538 (5)	
C3	−0.0787 (3)	0.6201 (2)	0.0817 (1)	0.0579 (5)	
C4	−0.1034 (4)	0.5384 (2)	0.1612 (1)	0.0546 (4)	
C5	0.0606 (3)	0.5473 (1)	0.2473 (1)	0.0454 (4)	
C6	0.2548 (3)	0.6396 (1)	0.2540 (1)	0.0388 (4)	
C7	0.4578 (3)	0.6773 (1)	0.3285 (1)	0.0400 (4)	
C8	0.5819 (3)	0.7783 (1)	0.2901 (1)	0.0466 (4)	
C9	0.5376 (3)	0.6213 (1)	0.4228 (1)	0.0411 (4)	
H1	0.524 (3)	0.865 (2)	0.159 (1)	0.062 (5)*	
H2	0.1210	0.7680	0.0330	0.065*	
H3	−0.1911	0.6111	0.0243	0.070*	
H4	−0.2329	0.4767	0.1561	0.065*	
H5	0.0419	0.4926	0.3002	0.054*	
H8	0.7215	0.8217	0.3228	0.056*	
H9	0.6619	0.6629	0.4650	0.049*	

### Atomic displacement parameters (Å^2^)

**Table:**

	*U*^11^	*U*^22^	*U*^33^	*U*^12^	*U*^13^	*U*^23^
N1	0.061 (1)	0.041 (1)	0.043 (1)	−0.002 (1)	0.005 (1)	0.012 (1)
N2	0.063 (1)	0.036 (1)	0.027 (1)	−0.001 (1)	−0.003 (1)	0.001 (1)
C1	0.049 (1)	0.039 (1)	0.039 (1)	0.006 (1)	0.006 (1)	0.005 (1)
C2	0.062 (1)	0.058 (1)	0.040 (1)	0.007 (1)	−0.001 (1)	0.014 (1)
C3	0.060 (1)	0.065 (1)	0.047 (1)	0.005 (1)	−0.008 (1)	0.003 (1)
C4	0.054 (1)	0.050 (1)	0.059 (1)	−0.003 (1)	−0.001 (1)	0.000 (1)
C5	0.052 (1)	0.040 (1)	0.045 (1)	0.003 (1)	0.006 (1)	0.004 (1)
C6	0.046 (1)	0.035 (1)	0.036 (1)	0.007 (1)	0.007 (1)	0.003 (1)
C7	0.052 (1)	0.034 (1)	0.034 (1)	0.004 (1)	0.004 (1)	0.001 (1)
C8	0.059 (1)	0.040 (1)	0.041 (1)	−0.002 (1)	0.001 (1)	0.003 (1)
C9	0.056 (1)	0.036 (1)	0.032 (1)	−0.003 (1)	−0.001 (1)	−0.003 (1)

### Geometric parameters (Å, °)

**Table:**

N1—C8	1.351 (2)	C6—C7	1.440 (2)
N1—C1	1.381 (2)	C7—C8	1.370 (2)
N2—C9	1.283 (2)	C7—C9	1.432 (2)
N2—N2^i^	1.409 (2)	N1—H1	0.87 (2)
C1—C2	1.387 (2)	C2—H2	0.9300
C1—C6	1.412 (2)	C3—H3	0.9300
C2—C3	1.365 (2)	C4—H4	0.9300
C3—C4	1.393 (2)	C5—H5	0.9300
C4—C5	1.377 (2)	C8—H8	0.9300
C5—C6	1.393 (2)	C9—H9	0.9300
			
C8—N1—C1	109.2 (1)	N2—C9—C7	123.4 (1)
C9—N2—N2^i^	112.0 (1)	C8—N1—H1	126 (1)
N1—C1—C2	129.9 (1)	C1—N1—H1	125 (1)
N1—C1—C6	107.6 (1)	C3—C2—H2	121.4
C2—C1—C6	122.5 (2)	C1—C2—H2	121.4
C3—C2—C1	117.3 (2)	C2—C3—H3	119.2
C2—C3—C4	121.7 (2)	C4—C3—H3	119.2
C5—C4—C3	121.2 (2)	C5—C4—H4	119.4
C4—C5—C6	118.9 (1)	C3—C4—H4	119.4
C5—C6—C1	118.5 (1)	C4—C5—H5	120.5
C5—C6—C7	135.2 (1)	C6—C5—H5	120.5
C1—C6—C7	106.3 (1)	N1—C8—H8	124.8
C8—C7—C9	123.7 (1)	C7—C8—H8	124.8
C8—C7—C6	106.5 (1)	N2—C9—H9	118.3
C9—C7—C6	129.7 (1)	C7—C9—H9	118.3
N1—C8—C7	110.5 (1)		
			
C8—N1—C1—C2	−179.5 (2)	C2—C1—C6—C7	179.4 (1)
C8—N1—C1—C6	0.5 (2)	C5—C6—C7—C8	−178.6 (2)
N1—C1—C2—C3	−179.6 (2)	C1—C6—C7—C8	0.5 (2)
C6—C1—C2—C3	0.4 (2)	C5—C6—C7—C9	5.1 (3)
C1—C2—C3—C4	0.7 (3)	C1—C6—C7—C9	−175.9 (2)
C2—C3—C4—C5	−0.7 (3)	C1—N1—C8—C7	−0.2 (2)
C3—C4—C5—C6	−0.3 (2)	C9—C7—C8—N1	176.5 (1)
C4—C5—C6—C1	1.3 (2)	C6—C7—C8—N1	−0.2 (2)
C4—C5—C6—C7	−179.7 (2)	N2^i^—N2—C9—C7	−178.9 (1)
N1—C1—C6—C5	178.6 (1)	C8—C7—C9—N2	−169.5 (1)
C2—C1—C6—C5	−1.4 (2)	C6—C7—C9—N2	6.3 (3)
N1—C1—C6—C7	−0.6 (2)		

Symmetry codes: (i) −*x*+1, −*y*+1, −*z*+1.

### Hydrogen-bond geometry (Å, °)

**Table:**

*D*—H···*A*	*D*—H	H···*A*	*D*···*A*	*D*—H···*A*
N1—H1···N2^ii^	0.87 (2)	2.21 (2)	3.065 (2)	167 (2)

Symmetry codes: (ii) −*x*+1, *y*+1/2, −*z*+1/2.
